# Interface States and Interface-Bulk Correspondence of One-dimensional Hyperbolic Metamaterials

**DOI:** 10.1038/srep43392

**Published:** 2017-02-24

**Authors:** Ieng-Wai Un, Ta-Jen Yen

**Affiliations:** 1 Department of Materials Science and Engineering, National Tsing Hua University, Hsinchu, 30013, Taiwan; 2 Department of Materials Science Center For Nanotechnology, Materials Science, and Microsystems, National Tsing Hua University, Hsinchu, 30013, Taiwan

## Abstract

We investigate the interface state on one-dimensional hyperbolic metamaterial (1DHMM). Initially, we analyze the plasmonic band structure of binary 1DHMM and analytically determine its band crossing condition. Then, we scrutinize the existence of an interface state in the plasmonic band gap of 1DHMM on three types of interfaces: dielectric/1DHMM, metal/1DHMM, and 1DHMM/1DHMM. We find that the band crossing dramatically influences the existence of an interface state. We also show a rigorous relation between the existence of the interface state of 1DHMM in the plasmonic band gap and the wave admittance in the plasmonic band region. More importantly, this relation not only holds for binary 1DHMM but also can be generalized to any 1DHMM with inversion symmetry. We also characterize the interface state by the transverse spin angular momentum and reveal the transverse spin flipping of the interface state.

Hyperbolic metamaterials (HMMs), a set of artificially tailored materials whose dispersion appears exotically hyperbolic instead of conventional elliptical contour^1^,^2^,^3^, have been attracting attention because these materials provide a variety of fascinating optical properties, such as negative refraction^4^,^5^, enhanced Purcell effect^6^,^7^, nonlocal effect^8^,^9^, far-field optical hyperlens^10^,^11^,^12^ and anomalous scaling^13^. To date, many reported works regarding HMMs have focused on their opical bulk properties^4^,^5^,^6^,^7^,^8^,^9^,^10^,^11^,^12^,^13^, interface state of HMMs have also been reported based on the topological transition of HMMs^2^,^14^,^15^; yet, the exceptional electromagnetic response of HMMs actually depends on their interface properties. Usually, the surface states can be described by the complex wave admittance Y (inverse of wave impedance), i.e., the ratio of the transverse magnetic field to the transverse electric field. For example, an electromagnetic (EM) wave can perfectly transmit through an interface between two media with the same wave admittance (*Y*_1_ = *Y*_2_). In addition, for the radiated wave, perfect transmission of the TM polarized EM waves at the Brewster angle can also be considered wave admittance matching in two media. Another example is the evanescent wave, the super-oscillating spatial frequency results in the exponential decay of the field intensity from the interface. In this case, the vanishing of the total wave admittance (*Y*_1_ + *Y*_2_ = 0) in two media inferred the existence of propagating interface state on the interface. For example, the condition *Y*_*d*_ + *Y*_*m*_ = 0 essentially determines the dispersion relation of the surface plasmon polariton (SPP) on the interface between dielectric and metal^16^. Thus, the wave admittance establishes the type of wave that propagates in a material and the material that attaches to form the interface state.

In addition, the interface property of 1DHMMs is expected to correspond to their bulk property. The “interface-bulk correspondence” refers to the existence of a protected surface state on an insulator due to the nontrivial topology of the band structure known as a topological insulator in electronic system^17^,^18^,^19^. The protected surface state appears unless the symmetry is broken or the band gap is closed. The existence of zero-energy edge state is topologically related to the bulk properties and the chiral symmetry^20^. Recently, topological insulators in photonic systems in analogy to the electronic system have been theoretically predicted and experimentally realized^15^,^21^,^22^,^23^,^24^,^25^,^26^,^27^,^28^,^29^. For example, unidirectional photonic edge states have been demonstrated by introducing magneto-optical effects^22^,^23^,^24^ and chirality^15^. By harmonically modulating the coupling constant within a resonator lattice, the effective magnetic field for a photon emerges and leads to a one-way photonic edge state without magneto-optical effects^29^. Magnetic topological transition between elliptic and hyperbolic iso-frequency contour has also been demonstrated in 2D transmission line metamaterials^30^ by changing the sign of admittance which is proportional to the effective premeability. For a periodic photonic system, the existence of an interface state may be related to the band structure in terms of the Zak phase^25^. For the photonic system studied by M. Xiao *et al*.^25^, the set of the photonic band gaps and the interface state are fixed by the thickness and dielectric constant of the composite layer up to some frequency shift.

In this article, we develop a rigorous interface-bulk correspondence that directly relates the existence of the interface state on 1DHMM in the plasmonic band gap with the wave admittance in the band region. Instead of effective medium theory, we adopt the transfer matrix method^31^ to calculate the plasmonic band structure of 1DHMM. In the plasmonic band gap, we investigate the formation of dielectric/1DHMM, metal/1DHMM and 1DHMM/1DHMM and their dispersion relations. By closing and reopening the band gap of a 1DHMM, we demonstrate that the required attaching material for interface state formation changes from a metallic to dielectric material (or vice versa). In other words, the band crossing significantly changes the existence of the interface state. More significantly, one can close and reopen the plasmonic band gap by altering the transverse wave vector and then modify the existence of the interface state in a single 1DHMM. We further show that this interface-bulk correspondence remains valid for any 1DHMM with inversion symmetry. We also analyze the optical spin angular momentum of the interface states to verify the interface-bulk correspondence.

## Results

**Figure 1:**
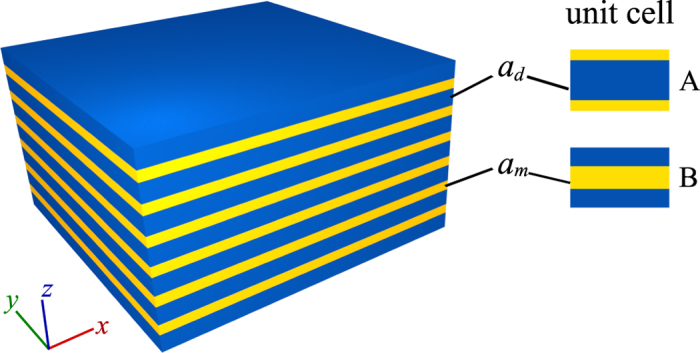
Schematics of a binary hyperbolic metamaterial. Hyperbolic metamaterial comprises alternating metal and dielectric layer of thickness *a*_*m*_ and *a*_*d*_, dielectric constants *ε*_*m*_ and *ε*_*d*_, respectively. We can choose the unit cell of binary system centered with inversion center which results in two kinds of unit cell A and B.

**Figure 2:**
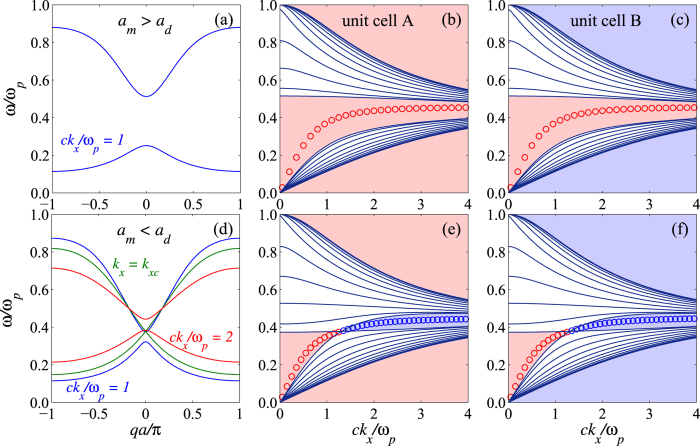
Plasmonic band structure of the binary 1DHMM. Plasmonic band structure of the binary 1DHMM with dielectric *ε*_*d*_ = 4 and metal 
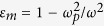
 but different thickness: (**a**–**c**) for *a*_*d*_ = 0.4 and *a*_*m*_ = 0.6 which leads to band structure without crossing; (**d**–**f**) for *a*_*d*_ = 0.6 and *a*_*m*_ = 0.4 which leads to band crossing. The bulk and gap properties shown in (**b**,**c**,**e**,**f**) correspond to the choice of unit cells A and B, respectively. In fact, the bulk band dispersion does not depend on the choice of unit cell but the interface property does (see the description in the text). The color in the gap depicts the required material type for interface state formation: red for dielectric and blue for metallic. Red circles show the dispersion of the interface state between the dielectric material (*ε*_*K*_ = 3) and the 1DHMM. Blue circles show the dispersion of the interface state between the metallic material 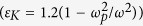 and the 1DHMM.

First, we restrict our attention to a binary 1DHMM comprised of alternative layers of metal and dielectric of thickness *a*_*m*_ and *a*_*d*_, as shown in Fig. 1. Moreover, the dielectric constant of the metal and dielectric are 
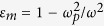
 and *ε*_*d*_, respectively, where *ω*_*p*_ denotes the plasma frequency. By solving the eigen-problem of the unit cell transfer matrix, we obtain two types of eigenvalues *λ*:


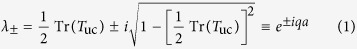


if |Tr(*T*_uc_)| ≤ 2, which corresponds to the band region and


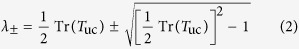


if |Tr(*T*_uc_)| > 2, which corresponds to the gap region, where *a* = *a*_*m*_ + *a*_*d*_ is the lattice constant. As a result, the band dispersion of binary 1DHMM is determined by


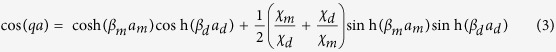


where 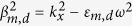, *χ*_*m,d*_ = *ε*_*m,d*_*ω/β*_*m,d*_, *k*_*x*_ is the transverse wave vector and *q* is the Bloch wave vector. The resulting band structure is shown in Fig. 2. One can verify that the band crossing occurs when


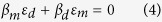



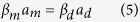


From the band crossing conditions Eq. (4) and Eq. (5), we can conclude that there is no band crossing for *a*_*d*_ < *a*_*m*_, see Fig. 2(a), and the band crossing (denoted as 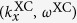) occurs at band center only when *a*_*d*_ > *a*_*m*_ (see Fig. 2(d)) regardless of the dielectric constants *ε*_*m*_ and *ε*_*d*_. One also requires that *ε*_*m*_ < 0, *ε*_*d*_ > 0 and 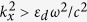 to fully satisfy these conditions.

To study the interface state, we attach a neighboring material of dielectric constant *ε*_*K*_ to semi-infinite 1DHMM terminated with a unit cell A or B. Similar to the interface states in other system (e.g., SPP), one expects an exponential decay in field intensity on both sides of the material. Thus, the eigenvalue of corresponding interface state of 1DHMM should satisfy |*λ*| < 1 and 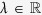 which is allowed in the gap region. According to our definition of the transfer matrix, the ratio of the two components in the eigenvector is exactly the admittance on the boundary of the unit cell, i.e., *Y*_*HMM*_ = *T*_uc,12_/(*λ* − *T*_uc,11_) or *Y*_*HMM*_ = (*λ* − *T*_uc,22_)/*T*_uc,21_, in both band and gap regions. Alternatively, the wave admittance in material K is found to be *Y*_*K*_ = −*ε*_*K*_*ω/β*_*K*_. By applying the interface existence condition *Y*_*HMM*_ + *Y*_*K*_ = 0, one can clearly see that the sign of *Y*_*HMM*_ determines the material type of K required to form an interface state. The next step requires the determination of sgn(*Y*_*HMM*_). For the unit cell with inversion symmetry, the unit cell transfer matrix has the property *T*_uc,11_ = *T*_uc,22_ = (1/2)Tr(*T*_uc_). Together with the requirement of |*λ*_gap_| < 1, one can prove that (*λ* − *T*_uc,11_) < 0(>0) for the gap between two band centers (edges). For a binary 1DHMM, we are interested in the gap between the band centers where band crossing occurs. For the case of *a*_*d*_ < *a*_*m*_, i.e., without band crossing, *T*_uc,12_ < 0 within the entire gap. Therefore, on one hand, interface state formation requires *ε*_*K*_ > 0, i.e., a dielectric, and the 1DHMM is said to be metallic-like (see Fig. 2(b) and (c)). On the other hand, for the case of *a*_*d*_ > *a*_*m*_, i.e., with band crossing, *T*_uc,12_ < 0 for 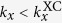 and the 1DHMM is metallic-like; when 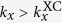, *T*_uc,12_ > 0 then the interface exists for the material K with negative permittivity and the 1DHMM is said to be dielectric-like (see Fig. 2(e) and (f)). The interface state can occur on the interface between dielectric-like and metallic-like 1DHMM. Remarkably, a single binary 1DHMM with *a*_*d*_ > *a*_*m*_ exhibits phase transition like behavior in the plasmonic band gap when the external transverse wave momentum is fine tuned around the band crossing point. We attribute its phase transition-like behavior to the dispersion of the metallic layer. In fact, the band crossing condition Eq. (4) is merely the dispersion relation of SPP on the interface between *ε*_*d*_ and *ε*_*m*_^16^. Therefore, the interface state and phase transition-like behavior can be experimentally realized without changing material or structural configuration.

**Figure 3:**
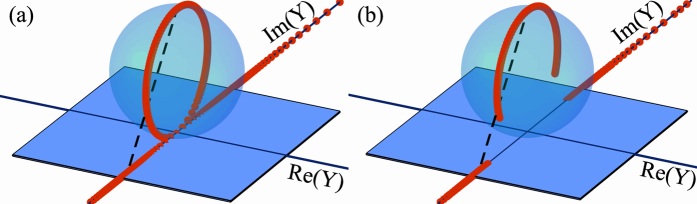
Complex wave admittance on the Riemann sphere. Stereographic projection of the complex wave admittance (red dot) on the Riemann sphere for *q* going a round trip on the Brillouin zone (for example, from −*π/a* to *π/a*). (**a**) If the number of singularities in the eigenvector of the unit cell transfer matrix is one, the wave admittance goes from −*i*∞ to *i*∞; when projected on the Riemann sphere, the path corresponds to a closed loop. (**b**) If the number of singularities is 0 or 2, the wave admittance projected on the Riemann sphere follow a retracted path.

Next, we devote the remainder of this Letter to the so called “interface-bulk correspondence” of 1DHMM with inversion symmetry. Recently, interface-bulk correspondence has been found in a 1D photonic crystal^25^ in terms of the Zak phase^32^ of the photonic band structure. The existence of interface state is determined by the formation condition *T*_*HMM*_ + *Y*_*K*_ = 0 and the wave admittance is position dependent in the periodic structure. It is interesting to search for interface-bulk correspondence in terms of wave admittance directly. In analogy to the Zak phase, we formally define


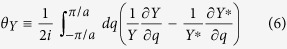


in our dispersive system. Recall that the wave admittance is equal to the ratio of the two components in eigenvector. In some sense, the integration in Eq. (6) involves counting the number of discontinuity of *Y*_*HMM*_ which is equivalent (mod2) to the number of singularity of the eigenvector within a specific band. Here, singularity refers to simultaneous zeros of two components of the eigenvector. For a unit cell with inversion symmetry, *Y(q*) = −*Y*(−*q*), the integration in Eq. (6) vanishes if *Y(q*) is continuous. However, if there are discontinuities at, say, *q*_*j*_’s and *Y* is (mathematically) discontinuous and Eq. (6) becomes


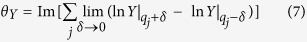


with inversion symmetry, *λ*_band_ − *T*_uc,11_ = *λ*_band_ − *T*_uc,22_ = *i* sin(*qa*), the singularity of the eigenvector may hence occur at the band center (*q *= 0) and/or band edge (*q* = ±*π/a*) only, depending on the zeros of *T*_uc,12_ and *T*_uc,21_. One can move the singularity from *q* = 0 to *q* = ±*π* (or vice versa) or annihilate them with each other if there are two, by gauge transformation on the eigenvector. If we take a closer look at the band edge or band center where *Y(q*) → ±*i*∞, the transverse magnetic field is finite while the transverse electric field is zero. So *Y(q*) → *i*∞ and *Y(q*) → −*i*∞ are different from each other in a irrelevant global phase, they should be regarded as the same state of the EM field. The same argument can be applied for the point where *Y(q*) → 0 likewise. Note that the global phase is irrelevant only when one of the traverse field is zeros. So the mathematical discontinuity of *Y(q*) stems from the usage of single function to represent *Y(q*) over the Brillouin zone. To illustrate the above concept, stereographic projection is introduced to map the wave admittance on the complex plane onto the Riemann sphere. Under the stereographic projection, we compactify the wave admittance on the complex plane (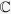) together with {∞}. By doing so, the lattice wave vector *q* in the 1D Brillouin zone (*S*^1^) is mapped to a unit circle (*S*^1^) on the Riemann sphere. Accordingly, on the other hand, if there is one singularity in the band, *θ*_*Y*_ = ±*π* and *Y* takes the form of a closed loop on the Riemann sphere, see Fig. 3(a). On the other hand, if there are two singularities (or none of them), *θ*_*Y*_ = 0 and *Y* takes a retracted path, see Fig. 3(b). Now we consider the connection between *θ*_*Y*_ and interface state existence. If we consider a specific band with *θ*_*Y*_ sandwich between two gaps. *T*_uc,11_ of these two gaps should have different sign because one of them is lying between band centers and the other is lying between band edges. And the zeros of *T*_uc,12_ or *T*_uc,21_ at the singularity imply sign flipping in these quantities. We can conclude that if *θ*_*Y*_ = ±*π (θ*_*Y*_ = 0), *T*_uc,12_ and *T*_uc,21_ of these gaps will have the different (same) sign, but the wave admittance will have the same (different) sign; hence, the interface states of 1DHMM in these gaps form with the same (different) material type. We emphasize that the “interface-bulk correspondence” in terms of wave admittance relies on the inversion symmetry only, i.e., it is not only limited to binary 1DHMM but also true for any 1DHMM with inversion symmetry.

**Figure 4:**
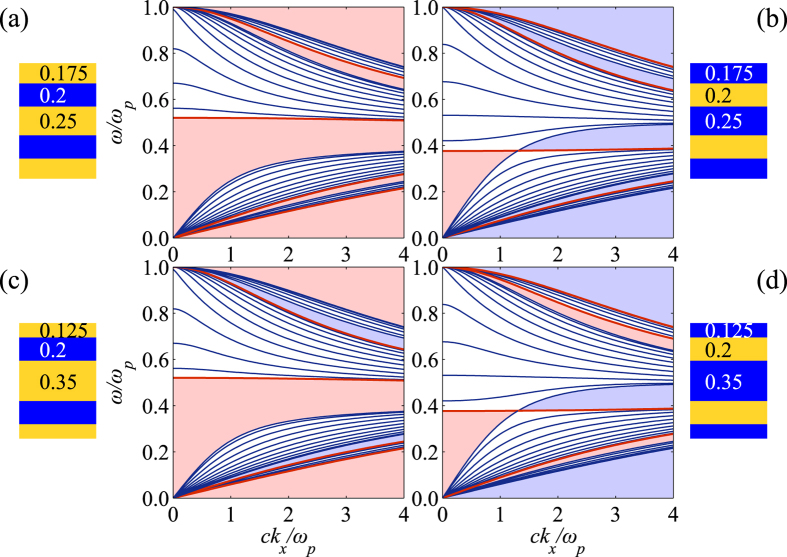
Band structure of the 1DHMM comprised of four layers in a unit cell. The unit cells are chosen as shown to preserve the inversion symmetry. The number on each layer denotes its thickness. Blue lines show the plasmonic band of 1DHMM. The red lines highlight the singularity of the eigenvector. Red and blue colors covering on the gaps denotes the required material for interface state formation as dielectric and metal, respectively. These two unit cells shown in (**a**,**c**) (also (**b**,**d**)) corresponding to the same 1DHMM but two gaps require different type of material for interface state formation.

Figure 4 shows the plasmonic band structure of a 1DHMM with a unit cell composed of four layers. In order to preserve, we choose the the unit cells as shown on the right hand side of the band structures. One can verify the “interface-bulk correspondence” by counting the number of singularities (highlighted by the red lines) of a specific band. Notice that these two unit cells generate the same bulk properties but different interface properties of 1DHMM. Semi-infinite HMM ended with these unit cells demand different types of materials for interface state formation in two gaps. A similar phenomenon occurs in the case of a binary 1DHMM as shown in Fig. 2 (although these gaps may not be interesting to researchers in practical application). Consequently, the wave admittance does play a significant role in connecting the interface and bulk properties.

## Discussion

We consider the detail of the binary 1DHMM based on the band crossing condition Eq. 5, i.e. *β*_*m*_*a*_*m*_ = *β*_*d*_*a*_*d*_. The plasmonic band structure of 1DHMM in fact results from the coupling of the surface plasmon on each interface between the dielectric and the metal. Therefore, we can regard the binary 1DHMM as the nearest neighbor tight binding model because of the exponentially decaying nature of the surface plasmons. The coupling constant can be characterized by the dimensionless quantity *β*_*i*_*a*_*i*_, *i* = *m* or *d*, where *β*_*i*_ is the field decay rate in material *i*. In this regard the binary 1DHMM shows a close analogy to the Su-Schrieffer-Heeger (SSH) model of polyacetylene^33^. The SSH model describes the behavior of a spinless Fermion in a conjugated polymer with staggered hopping amplitude *t*_1_ and *t*_2_. Near *t*_1_ = *t*_2_, the occurrence of topological phase transition accompanies the closing and reopening of the energy gap. Similar to the SSH model, the closing and reopening of plasmonic band gap in binary 1DHMM arises from the changing of *θ*_*Y*_ near *β*_*m*_*a*_*m*_ = *β*_*d*_*a*_*d*_. By altering the transverse momentum *k*_*x*_, we are substantially varying the coupling strength between surface plasmons on neighbor interfaces, revealing phase transition and interface state formation with different types of material.

We further analyze the interface state around the band crossing point by the transverse spin angular momentum. Transverse optical spin angular momentum (SAM) have been recently discovered in the evanescent wave^34^, two interference waves^35^ and the surface waves^36^. Particularly, the evanescent wave and surface waves exhibit universal transverse spin-momentum locking feature^37^ which leads to interface states of photonic topological insulator^38^. On the other hand, the electromagnetic waves propagating in the 1DHMM are essentially coupled plasmon on each interface between metal and dielectric layer. We show that the interface states of 1DHMM exhibit strong spin-momentum locking and the transverse spin flipping during the phase transition which demanding different material for interface state formation. Applying Noether theorem to the electromagnetic field Langrangian respect to the spatial translational symmetry and rotational symmetry, one can obtain the total optical momentum and angular momentum density of canonical form, respectively^36^,^39^. Rewrite the optical momentum and angular momentum density into dual symmetric form according to the discrete dual symmetry, then the optical spin angular momentum density can be identified as the difference between the total angular momentum density and the orbital part. In the monochromatic limit, the spin angular momentum density reads^39^


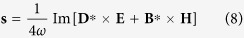


**Figure 5:**
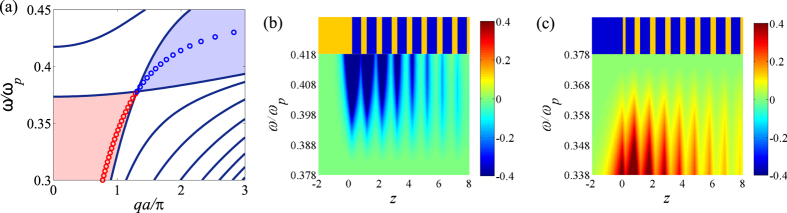
Optical spin angular momentum of the interface state. We attach a dielectric *ε*_*d*_ = 4 (and metallic 
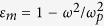
) material to the 1DHMM with *ε*_*d*_ = 4, 
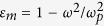
, *a*_*d*_ = 0.6 and *a*_*m*_ = 0.4 to form interface state before (and after) the band crossing. We calculate the transverse spin angular momentum of the interface state along the dispersion relation. The transverse SAM flips when the interface passes the band crossing point at *ω/ω*_*p*_ = 0.387.

For the TM polarization, only the electric part contributes to the spin angular momentum. The eigenvectors of the transfer matrix allow us to calculate the transverse SAM density explicitly. We follow the procedure in the preceding section to calculate the dispersion relation and eigenvectors of the interface state forming with dielectric (and metal) before (and after) the band crossing respectively. Figure 5 shows the transverse SAM density of the interface state in the heterostructure along the dispersion relation. We can compare the SAM density of interface state on the HMM with the SAM density of SPP as shown in Fig. S7 in the Supplementary Information. Consider the interface state and SPP propagating in the +*x* direction, for the case of Fig. 5(c) *k*_*x*_ < *kx*^*XC*^, the transverse SAM is locked to the +*y* direction, similar to the case of Fig. S7(b) and (d) in the Supplementary Information where the metal locates at the right hand side. On the other hand, for the case of Fig. 5(b) 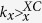, the transverse SAM is locked to the +*y* direction, similar to the case of Fig. S7(a) and (c) in the Supplementary Information. One can clearly see that the SAM flips its direction when the interface state passes the band crossing point. The transverse SAM flipping in the HMM indicates the phase transition like behaviour and requirement of different material for interface state formation simultaneously. The spin-momentum locking feature also provides the potential opportunity to demonstrate unidirectional interface state excitation.

The robustness of the interface state suffers from any disorder which breaks the inversion symmetry or uniformity. Disorders locally alternate the interface state formation condition and result in scattering of the interface state. Common disorders in HMM are roughness and non-uniformity, which can be regarded as additional scattering loss and do not eliminate the existence of the interface state but increase the spectral linewidth when the interface state is excited.

## Conclusion

We investigated the interface state of 1DHMM, and demonstrated that near the band crossing, the interface changes significantly from metallic-like to dielectric-like property. We also demonstrated the “interface-bulk correspondence” directly in terms of wave admittance and that such a result is valid for any type of 1DHMM with inversion symmetry. In addition, we analytically showed the band crossing condition for binary 1DHMM and its close analogy to the topological phase transition in the SSH model. We also analysis the transverse spin angular momentum of the interface states and show that the transverse SAM flips around the band crossing point. With these findings aforementioned, one can close and reopen the plasmonic band gap of 1DHMM by tuning the transverse momentum and manifest the phase transition-like property in a single 1DHMM without changing the material or structural properties.

## Methods

The transfer matrix *T*(Δ*z*) is defined by transforming the transverse EM field by a distance Δ*z*


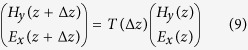


where


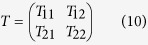


and det*T* = 1. One can choose the center of the unit cell coinciding with the inversion center, which leads to two types of unit cells with unit cell transfer matrices 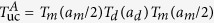 and 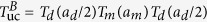. Due to the periodic nature of the 1DHMM, the propagating EM fields should satisfy the Bloch solution.

## Additional Information

**How to cite this article:** Un, I.-W. and Yen, T.-J. Interface States and Interface-Bulk Correspondence of One-dimensional Hyperbolic Metamaterials. *Sci. Rep.* **7**, 43392; doi: 10.1038/srep43392 (2017).

**Publisher's note:** Springer Nature remains neutral with regard to jurisdictional claims in published maps and institutional affiliations.

## Supplementary Material

Supplementary Information
