# Supplementary material for: Interface States and Interface-Bulk Correspondence of One-dimensional Hyperbolic Metamaterials
Source: Sci Rep. 2017 Feb 24;7:43392. doi: 10.1038/srep43392 (PMC5324073; doi:10.1038/srep43392)
Supplement: Supplementary Information [file srep43392-s1.pdf]

Supplementary Information - Interface States and Interface-Bulk Correspondence of One-dimensional Hyperbolic Metamaterials

Ieng-Wai Un[1] and Ta-Jen Yen[1,2,*]

[1]Department of Materials Science and Engineering, National Tsing Hua University, Hsinchu, 30013, Taiwan
[2]Department of Materials Science Center For Nanotechnology, Materials Science, and Microsystems, National Tsing Hua University, Hsinchu, 30013, Taiwan
[*]tjyen@mx.nthu.edu.tw

December 2016

1 Admittance and Interface State

Consider the TM polarized interface state, the electromagnetic (EM) fields of an interface state in a semi-infinite material is assumed to be

$$\mathcal{H}_y(x,z,t) = H_y e^{ik_x x}\sin(\omega t) \quad \text{and} \quad \mathcal{E}_x(x,z,t) = E_x e^{ik_x x}\cos(\omega t)$$

$$H_y = Be^{-\beta z} \quad \text{and} \quad E_x = -\frac{\beta}{\epsilon\omega}Be^{-\beta z}$$

where $\beta^2 = k_x^2 - \epsilon\omega^2$, k_x is the transverse wave vector. So the admittance is found to be $Y = -\frac{\epsilon\omega}{\beta}$. On the other hand, the formation of interface state requires matching the boundary condition $\frac{\epsilon_1}{\beta_1} + \frac{\epsilon_2}{\beta_2} = 0$ which is equivalent to $Y_1 + Y_2 = 0$.

2 Plasmonic band structure of 1DHMM

We assume the EM fields in each layer to be

$$\begin{aligned} H_y &= Ae^{\beta(z-z_R)} + Be^{-\beta(z-z_L)} \\ E_x &= \frac{\beta}{\epsilon\omega}\left(Ae^{\beta(z-z_R)} - Be^{-\beta(z-z_L)}\right) \end{aligned}$$

where $z_L(z_R)$ is the left(right) side of the layer. The transfer matrix that relates the fields between the left and right sides can be written as

$$\begin{pmatrix} H_y \\ E_x \end{pmatrix}_{z_R} = \begin{pmatrix} \cosh(\beta d) & \frac{\epsilon\omega}{\beta}\sinh(\beta d) \\ \frac{\beta}{\epsilon\omega}\sinh(\beta d) & \cosh(\beta d) \end{pmatrix} \begin{pmatrix} H_y \\ E_x \end{pmatrix}_{z_L} \tag{S1}$$

where $d = z_R - z_L$ is the thickness of the layer. So we can determine the transfer matrix of the unit cell T_{uc} accordingly. Notice that the determinant of the transfer matrix is equal to 1 and if we choose the center of the unit cell coincides with the inversion center, $T_{uc,11} = T_{uc,22} = \frac{1}{2}\mathrm{Tr}(T_{uc})$. The eigenvalues of the transfer matrix are given by

$$\lambda_\pm = \frac{1}{2}\mathrm{Tr}(T_{\mathrm{uc}}) \pm i\sqrt{1 - \left[\frac{1}{2}\mathrm{Tr}(T_{\mathrm{uc}})\right]^2} \equiv e^{\pm iqa} \tag{S2}$$

if $|\mathrm{Tr}(T_{\mathrm{uc}})| \leq 2$, which corresponds to the band region and

$$\lambda_\pm = \frac{1}{2}\mathrm{Tr}(T_{\mathrm{uc}}) \pm \sqrt{\left[\frac{1}{2}\mathrm{Tr}(T_{\mathrm{uc}})\right]^2 - 1} \tag{S3}$$

if $|\mathrm{Tr}(T_{\mathrm{uc}})| > 2$, which corresponds to the gap region. The corresponding eigenvectors are given by

$$v_{\pm} = \begin{pmatrix} T_{uc,12} \\ \lambda_{\pm} - T_{uc,11} \end{pmatrix} \quad \text{or} \quad v_{\pm} = \begin{pmatrix} \lambda_{\pm} - T_{uc,22} \\ T_{uc,21} \end{pmatrix} \tag{S4}$$

in both the gap and band region. These two pair of eigenvectors relate to each other by gauge transformation. The unit cell transfer matrix relates the transverse EM fields at the both side of the unit cell, so the admittance is equal to the ratio of the two components of the eigenvector, i.e. $Y_{HMM} = T_{\mathrm{uc},12}/(\lambda - T_{\mathrm{uc},11})$ (or $Y_{HMM} = (\lambda - T_{\mathrm{uc},22})/T_{\mathrm{uc},21}$).

3 Interface states of 1DHMM and the interface-bulk correspondence

To determine the interface state of 1DHMM, we attach the 1DHMM to another material K with dielectric constant ϵ_K and admittance $-\frac{\epsilon_K \omega}{\beta_K}$. Similiarly, the formation of interface state requires $Y_{HMM} + Y_K = 0$. Thus, the sign of Y_{HMM} in the band gap determines the sign of ϵ_K to form interface state: $\epsilon_K < 0$ if $Y_{HMM} > 0$ ($\epsilon_K > 0$ if $Y_{HMM} < 0$). Now we are going to determine the signs of the two components of the eigenvector and prove the interface-bulk correspondence with the inversion symmetry by assuming that all the components of the transfer matrix are continuous. Recall that (a) $T_{uc,11} = T_{uc,22} = \frac{1}{2}\mathrm{Tr}(T_{uc})$ for inversion symmetry. (b) $\det(T_{uc}) = T_{uc,11}T_{uc,22} - T_{uc,12}T_{uc,21} = 1$. (c) $\frac{1}{2}\mathrm{Tr}(T_{uc}) = \cos(qa)$ in band region and $|\frac{1}{2}\mathrm{Tr}(T_{uc})| > 1$ in the gap region. As a result, at least one of $T_{uc,12}$ and $T_{uc,21}$ is equal to zero at the band center ($\cos(qa) = 1$) and band edge ($\cos(qa) = -1$). We first find out the sign of $\lambda - T_{uc,11} = \lambda - \frac{1}{2}\mathrm{Tr}(T_{uc}) = \pm\sqrt{[\frac{1}{2}\mathrm{Tr}(T_{uc})]^2 - 1}$. For a specific gap between two band centers (band edges), we have $\frac{1}{2}\mathrm{Tr}(T_{uc}) > 1$ ($\frac{1}{2}\mathrm{Tr}(T_{uc}) < -1$). Combining with $|\lambda| < 1$ in gap region, a requirement for the formation of interface state whose EM fields exponentially decay from the interface, we conclude that $\lambda - T_{uc,11} < 0$ ($\lambda - T_{uc,11} > 0$). Then we are going to find the sign of $T_{uc,12}$. The sign of $T_{uc,12}$ at the gap region depends on its value at the band region. Consider a band between two gaps, if $T_{uc,12} = 0$ at the band edge or band center but not both, $T_{uc,12}$ will have different sign in these two gap; if $T_{uc,12} = 0$ at both or none of the band edge and band center, $T_{uc,12}$ will have same sign in these two gaps. On the other hand, $\lambda - T_{uc,11}$ have different sign in these two gaps according to the argument in the first step. Recall that the admittance of the HMM, Y_{HMM}, is the ratio of the two components of the eigenvector. Consequently, consider a band between two gaps, if $T_{uc,12} = 0$ at the band edge or band center but not both, Y_{HMM} will have same sign in these two gap, i.e. the HMM forms interface state with the materials with dielectric constant of the same sign in these two gaps; if $T_{uc,12} = 0$ at both or none of the band edge and band center, Y_{HMM} will have different sign in these two gap, i.e. the HMM forms interface state with the materials with dielectric constant of the different sign in these two gaps. These conclusions are indeed guaranteed by the conditions (a), (b), (c) and the continuity of the components of the unit cell transfer matrix.

In order to identify the interface-bulk correspondence in terms of the admittance, we are going to determine the admittance at the band center and band edge, only where the singularity of the eigenvector occurs. The singularity refers to the simultaneous zeros of the two components of the eigenvector. Due to the inversion symmetry, $T_{uc,12}(q)$ is a symmetric function of q, i.e. $T_{uc,12}(q) = T_{uc,12}(-q)$. So $(\partial T_{uc,12}(q)/\partial q)_{qa=0} = 0$ and $(\partial T_{uc,12}(q)/\partial q)_{qa=\pm\pi} = 0$. On the other hand, $\lambda - T_{uc,11} = i\sin(qa)$, As a result, the singularity of the eigenvector locates at the admittance of HMM with $\pm\infty$ (if we choose the other eigenvector, the singularity of the eigenvector locates at the admittance of HMM with 0). There are four possible results (up to a $\pm$ sign) of $T_{uc,12}(q)$ and admittance of HMM in the band region as shown in Supplementary Fig. S**1**. (i) $T_{uc,12}(qa = \pm\pi) \neq 0$ and $T_{uc,12}(qa = \pm\pi) \neq 0$; (ii) $T_{uc,12}(qa = \pm\pi) \neq 0$ and $T_{uc,12}(qa = \pm\pi) = 0$; (iii) $T_{uc,12}(qa = \pm\pi) = 0$ and $T_{uc,12}(qa = \pm\pi) \neq 0$; (iv) $T_{uc,12}(qa = \pm\pi) = 0$ and $T_{uc,12}(qa = \pm\pi) = 0$. The first column shows the four possible results of $T_{uc,12}(q)$ (up to a $\pm$ sign), the second column shows the corresponding admittance of HMM as function of qa, the third column shows the trajectory of the corresponding admittance in the complex plane (all the trajectories overlap with the imaginary axis, we insert the offset in order to show the trajectories clearly). We calculate the $\theta_Y = \frac{1}{2i}\int_{q/a=-\pi}^{q/a=\pi} dq \left(\frac{1}{Y}\frac{\partial Y}{\partial q} - \frac{1}{Y^*}\frac{\partial Y^*}{\partial q}\right) = \int_{q/a=-\pi}^{q/a=\pi} dq \frac{\partial \arg(Y)}{\partial q}$ and let $Y \to Y + \epsilon$ with $\epsilon \to 0^+$ in order to smooth the $\arg(Y)$: (i) $\theta_Y = (\arg(Y)|_{qa\to-\pi} - \arg(Y)|_{qa\to\pi}) + (\arg(Y)|_{qa\to 0^+} - \arg(Y)_{qa\to 0^-}) = (\pi/2) - (-\pi/2) + (-\pi/2) - (\pi/2) = 0$; (ii) $\theta_Y = (\arg(Y)|_{qa\to 0^+} - \arg(Y)|_{qa\to 0^-}) = -\pi$; (iii) $\theta_Y = (\arg(Y)|_{qa\to-\pi} - \arg(Y)|_{qa\to\pi}) = \pi$; (iv) $\theta_Y = 0$. Consequently, calculation of θ_Y equivalently counts the number of singularity of the eigenvector.

The discontinuity of the admittance at the band center or band edge is considered to be mathematical but not physical. As mentioned in the main text, both $Y = i\infty$ and $Y = -i\infty$ correspond to zero transverse electric field with finite transverse magnetic field, they differ in a global phase from each other and can be considered as the same state. So we compactify the complex plane ($\mathbb{C}$) together with $\{\infty\}$ such that the lattice wave vector q in the 1D Brillouin zone (S^1) is mapped to a unit circle (S^1) on the Riemann sphere. Accordingly, if there is one

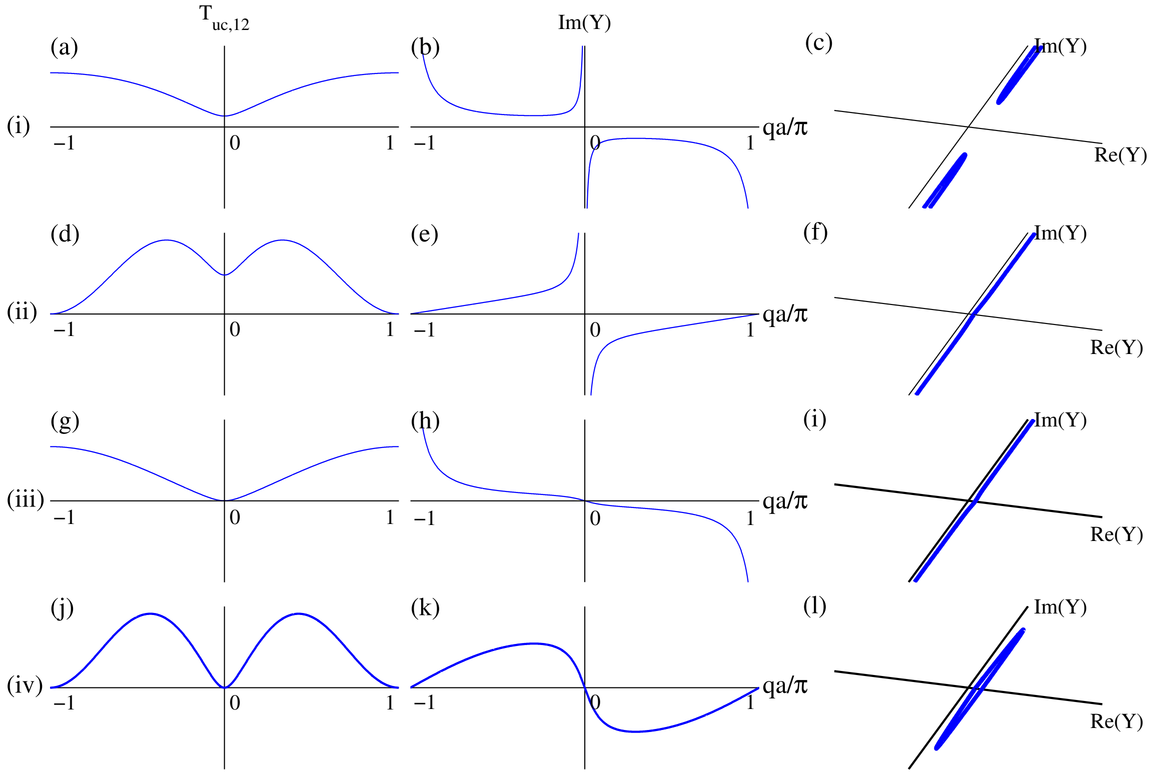

Supplementary Figure 1: **The admittance of the HMM in the band region.** The inversion symmetry indicates that there are four possible results of $T_{uc,12}$ (up to $\pm$ sign) as shown in the first column. The second column shows the corresponding admittance of the HMM in the band region. The third column shows the corresponding trajectory of admittance on the complex plane. By the compactification of the complex plane and stereographic projection, the corresponding trajectories on the Riemann sphere take close loop for case (ii) and (iii), retracted path for case (i) and (iv). So the interface state formation requires materials with dielectric constant of same (different) sign in the two gaps sandwiching the band for case (ii) and (iii) ((i) and (iv)).

singularity in the band, $\theta_Y = \pm\pi$ and Y takes the form of a closed loop on the Riemann sphere, i.e. case (ii) and (iii). On the other hand, if there are two singularities (or none of them), $\theta_Y = 0$ and Y takes a retracted path, i.e. case (i) and (iv). In summary, by considering a band between two gaps, we have first related the requirement for the interface state formation in the two gaps by characterizing the components of the eigenvector in the band, we have also shown that this relation can be interpreted by the admittance in the band region and we have further classified the trajectory of the admittance on the Riemann sphere accordingly: the number of zero of $T_{uc,12}$ is equal to the number of singularity of the eigenvector, $\theta_Y = \pm\pi$ if there is one singularity and $\theta_Y = 0$ if there is two or no singularity, the trajectory of the admittance on the Riemann sphere takes closed loop and retracted path, and the gaps switching the band form interface state with material with dielectric constant of the same and different sign, respectively.

Before we end this section, we would like to compare the usage of θ_Y and Zak phase to analyze the system including dispersive material. First of all, the interface state formation is directly determined by the condition $Y_{HMM} + Y_K = 0$ in the band gap region. That's why we want to relate the existence of interface state to the admittance in the band region. Second, by using the admittance approach we can derive the "interface-bulk correspondence" without knowing the detail dispersion of the material and the band structure HMM. Third, we can determine the dispersion of the interface state by the formation condition $Y_{HMM} + Y_K = 0$. On the other hand, the Zak phase[1] ϕ_{Zak} is expressed by the Bloch function $u_q(z) = e^{-iqz}\psi_q(z)$ which is normalized $\langle u_q|u_q\rangle = 1$.

$$\phi_{Zak} = i\int_{-\pi/a}^{\pi/a} \langle u_q|\,\partial_q\,|u_q\rangle\, dq \tag{S5}$$

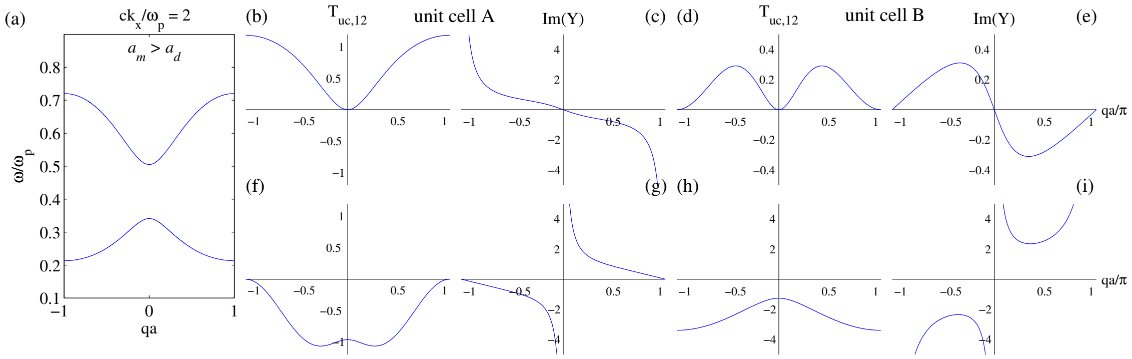

Supplementary Figure 2: **The band structure, $T_{ux,12}$ and admittance of binary HMM with $a_m/a_d = 3/2$, $ck_x/\omega_p = 2$.** (a) the band structure of the HMM. The upper and lower rows correspond to the upper and lower band, respectively. (b), (d), (f) and (h) show $T_{ux,12}$. (c), (e), (g) and (i) show imaginary part of the admittance and correspond to case (iii), (iv), (ii) and (i), respectively.

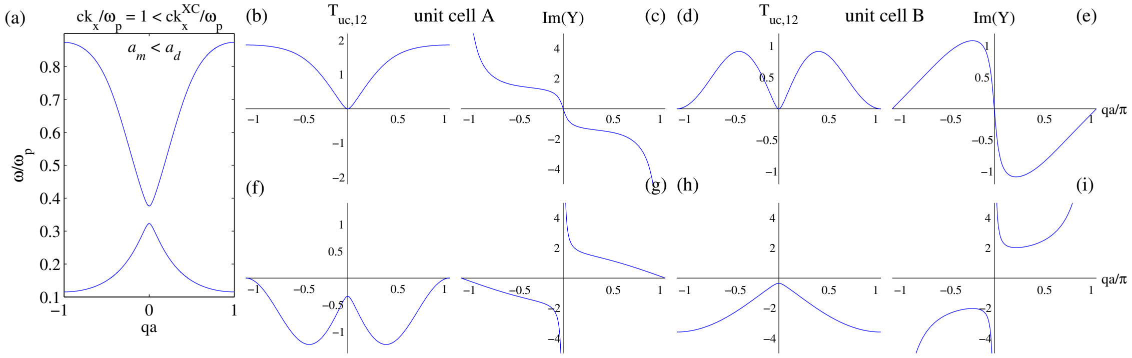

Supplementary Figure 3: **The band structure, $T_{ux,12}$ and admittance of binary HMM with $a_m/a_d = 3/2$, $ck_x/\omega_p = 1 < ck_x^{XC}/\omega_p$.** (a) the band structure of the HMM. The upper and lower rows correspond to the upper and lower band, respectively. (b), (d), (f) and (h) show $T_{ux,12}$. (c), (e), (g) and (i) show imaginary part of the admittance and correspond to case (iii), (iv), (ii) and (i), respectively.

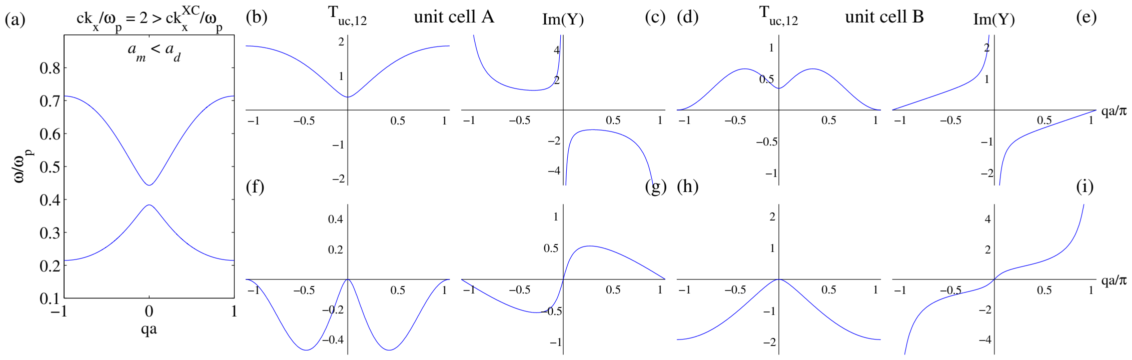

Supplementary Figure 4: **The band structure, $T_{ux,12}$ and admittance of binary HMM with $a_m/a_d = 2/3$, $ck_x/\omega_p = 2 > ck_x^{XC}/\omega_p$.** (a) the band structure of the HMM. The upper and lower rows correspond to the upper and lower band, respectively. (b), (d), (f) and (h) show $T_{ux,12}$. (c), (e), (g) and (i) show imaginary part of the admittance and correspond to case (i), (ii), (iv) and (iii), respectively.

For HMM, we can obtain the unnormalized Bloch function from the transfer matrix and its eigenvector. However, the normalization process will introduce cumbersome calculation in calculating the Zak phase. The cumbersomeness

comes from how to normalize the Bloch function correctly. Unlike the wavefunction in quantum mechanics, whose absolute square represents the probability of the particle, the components of the eigenvector of the transfer matrix represent the transverse electromagnetic (EM) fields. Therefore, in a unit cell one should normalize the Bloch function by unit energy, whose the electric part of the field energy[2,3] reads $\frac{d(\epsilon\omega)}{d\omega}|E|^2$ (similar for the magnetic part) for dispersive material, and the normalization constant is q or frequency dependent due to the band dispersion. On the other hand, one can convert the problem including dispersive material into a hermitian eigenvalue problem.[4] However, this conversion process requires the detail dispersion of the material. If we followed these approaches to calculate the Zak phase, we can, in principle, obtain the Zak phase after cumbersome calculation, but either the dispersion of the material or the band dispersion $q(\omega)$ of the HMM is required for calculating $\langle u_q| \partial_q |u_q\rangle$.

4 Examples

In this section, we will give some examples mentioned in the main text. For the binary 1DHMM, the band plasmonic band structure is determined by

$$\cos(qa) = \cosh(\beta_m a_m)\cosh(\beta_d a_d) + \frac{1}{2}\left(\frac{\chi_m}{\chi_d} + \frac{\chi_d}{\chi_m}\right)\sinh(\beta_m a_m)\sinh(\beta_d a_d) \tag{S6}$$

where $\chi_{m,d} = \epsilon_{m,d}\omega/\beta_{m,d}$ and q is the Bloch wave vector, where $a = a_m + a_d$ is the lattice constant. Supplementary Fig. S**2** and Supplementary Fig. S**3** show the band structure, $T_{ux,12}$ and admittance with $a_m/a_d = 3/2$, $ck_x/\omega_p = 2$ and that with $a_m/a_d = 2/3$, $ck_x/\omega_p = 1 < ck_x^{XC}/\omega_p$, respectively. For the case of unit cell A, the admittance of the upper and lower band are of case (iii) $\theta_Y = -\pi$ and (ii) $\theta_Y = \pi$, respectively. So all the three gaps require the material with dielectric constant of the same sign to form interface state, as shown in the Fig. 2(b) and Fig. 2(e) with $k_x < k_x^{XC}$. For the unit cell B, the admittance of the upper and lower band are of case (iv) $\theta_Y = 0$ and (i) $\theta_Y = 0$, respectively. So the middle gap requires material with dielectric constant of different sign from that required for the other two to form interface state, as shown in the Fig. 2(c) and and Fig. 2(f) with $k_x < k_x^{XC}$. On the other hand, Supplementary Fig. S**4** shows the band structure, $T_{ux,12}$ and admittance with $a_m/a_d = 2/3$, $ck_x/\omega_p = 2 > ck_x^{XC}$. For the case of unit cell A, the admittance of the upper and lower band are of case (i) $\theta_Y = 0$ and (iv) $\theta_Y = 0$, respectively. So the middle gap requires material with dielectric constant of different sign from that required for the other two to form interface state, as shown in the Fig. 2(e) with $k_x > k_x^{XC}$. For the unit cell B, the admittance of the upper and lower band are of case (ii) $\theta_Y = \pi$ and (iii) $\theta_Y = -\pi$, respectively. So all the three gaps require the material with dielectric constant of the same sign to form interface state, as shown in the Fig. 2(f) with $k_x > k_x^{XC}$.

5 Excitation of the interface state

The interface state of HMM can be excited based on the prism couping measurement. We will give an example of exciting the interface state of binary HMM inside the middle gap. The dispersion relation of the interface state depends on the dielectric constants ϵ_m, ϵ_d and ϵ_K according to $Y_{HMM} + Y_K = 0$. For the binary HMM, For $a_m > a_d$, the HMM form interface state with dielectric material. If we choose $\epsilon_K = \epsilon_d$, the dispersion of the interface state will be

$$k_x = \frac{\omega}{c}\sqrt{\frac{\epsilon_m\epsilon_d}{\epsilon_m + \epsilon_d}} \tag{S7}$$

which is exactly the dispersion of surface plasmon polariton between ϵ_m and ϵ_d. This result is also valid for $a_m < a_d$ with $k_x < k_x^{XC}$. On the other hand, for $a_m < a_d$ with $k_x > k_x^{XC}$, if we choose $\epsilon_K = \epsilon_m$, the dispersion of the interface state will also be $k_x = \frac{\omega}{c}\sqrt{\frac{\epsilon_m\epsilon_d}{\epsilon_m+\epsilon_d}}$, Moreover, the band crossing condition Eq (4) in the main text coincides with Eq. S7 and the band crossing frequency ω^{XC} decreases as decrease the ratio of a_m/a_d decreases according to Eq (5) in the main text.

Based on these properties, we will excite the interface state by a white light source with fixed incident angle and calculating the reflection with varying the ratio of a_m/a_d. Because the total thickness of the HMM is too thick for evanescent wave going through, we excite the interface state between dielectric and HMM by the Otto configuration as shown in Supplementary Fig. S**5** (c); interface state between metal and HMM by the Kretschmann (KR) configuration as shown in Supplementary Fig. S**5** (d). Starting from $a_m/a = 0.4$, the HMM forms interface state with dielectric material, so we can observe reflection deep in the spectrum. As a_m/a decreases, the band crossing frequency decreases, the HMM eventually form interface state with metal, then the reflection deep disappears

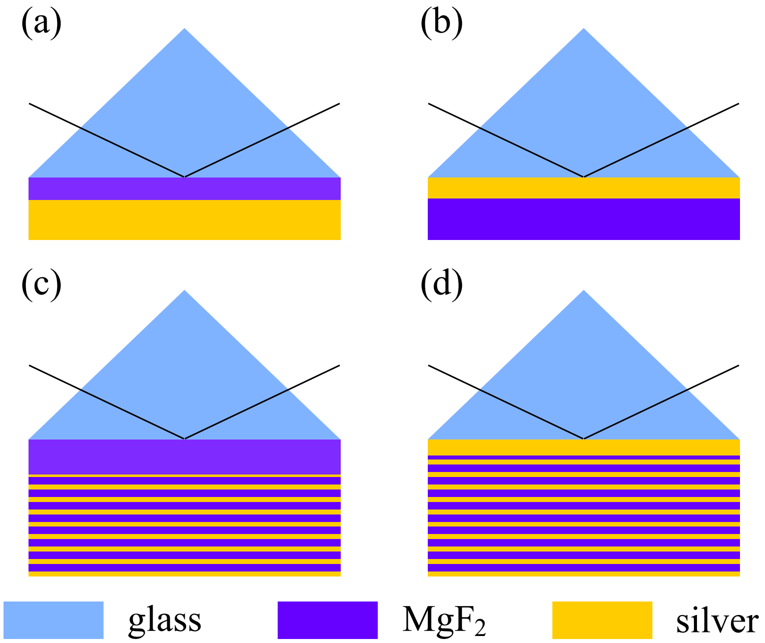

Supplementary Figure 5: **Excitation of the interface between three kind of interface: metal/dielectirc, metal/HMM, and dielectric/HMM.** The surface plasmon polariton between metal and dielectric can be excited by (a) the Otto or (b) Kretschmann (KR) configuration. (c) considering the thickness of the HMM, the interface state between HMM and dielectric is excited by the Otto configuration and (d) the interface state between HMM and metal is excited by the KR configuration.

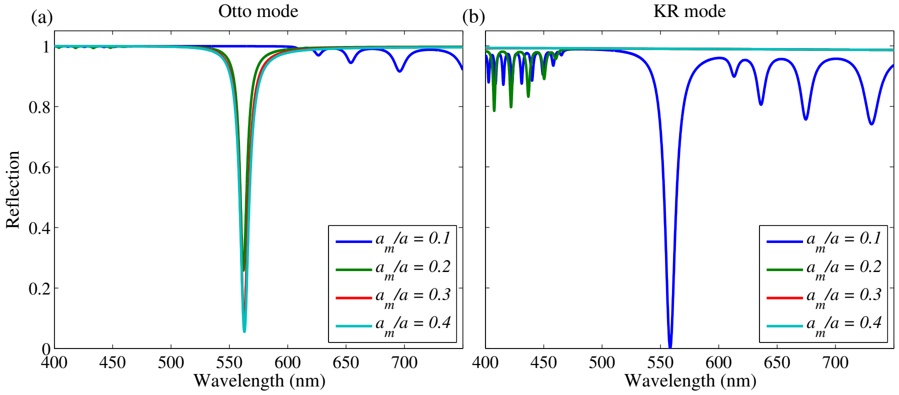

Supplementary Figure 6: **The reflection of the HMM under KR and Otto configuration.** (a) The interface state of HHM is excited by Otto configuration. For $a_m/a = 0.2$, 0.3 and 0.4, the HMM forms interface state with MgF_2 at the same wavelength but no interface state is excited for $a_m/a = 0.1$. (b) The interface state of HHM is excited by Otto configuration. For $a_m/a = 0.1$., the HMM forms interface state with silver but no interface state is excited for $a_m/a = 0.2$, 0.3 and 0.4.

in the Otto mode but appears in the KR mode. The critical ratio for binary HMM can be calculated to be $a_m/a = \frac{\epsilon_g \sin^2\theta - \epsilon_d}{2\epsilon_g \sin^2\theta - \epsilon_d}$, where ϵ_g is the dielectric constant of coupling prism.

In the calculation, we fix the incident angle by 80 degrees, the HMM comprises multilayer of silver and MgF_2. The dielectric constant of silver is numerically fitted by the Drude model $\epsilon_m = \epsilon_\infty \left(1 - \frac{\omega_p^2}{\omega(\omega+i\gamma)}\right)$ with $\epsilon_\infty = 3.7$, $\omega_p = 7.23 \times 10^{15} rad/s$ and $\gamma = 3.23 \times 10^{13} rad/s$. The dielectric constant of glass and MgF_2 are 1.5 and 1.37, respectively. The results are shown in Supplementary Fig. S**6**. The critical ration is 0.123. For $a_m/a = 0.2$, 0.3 and 0.4, the HMM form interface state with MgF_2 at the same wavelength (about 558 nm), so we can observe reflection deep in the Otto configuration as shown in Supplementary Fig. S**6** (a) but no reflection deep in the KR

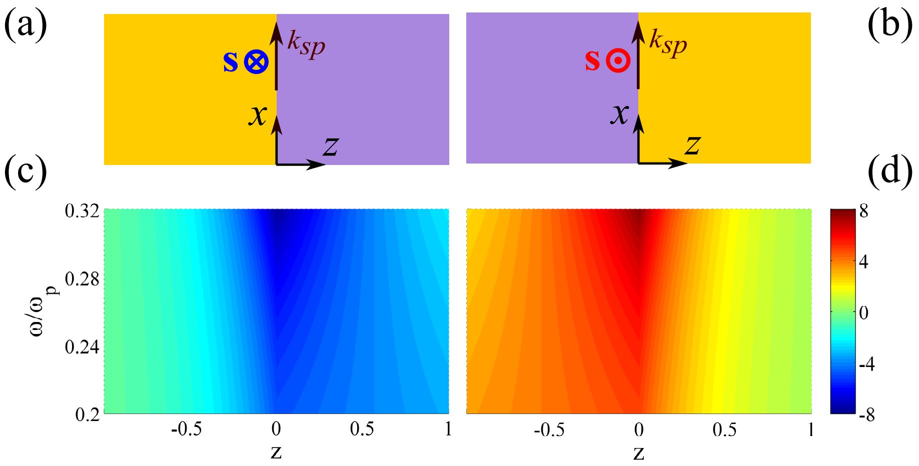

Supplementary Figure 7: **Transverse spin angular momentum density of surface plasmon.** The SAMs of the surface plasmon are calculated with $\omega/\omega_p = 0.2 \sim 0.32$ along its dispersion relation. (a) The surface plasmon propagates to the $+x$ direction on the interface between metal and dielectric. (c) the resulting SAM is locked to the $-y$ direction. (b) and (d) are mirror to (a) and (c), respectively.

configuration as shown in Supplementary Fig. S**6** (b). For $a_m/a = 0.1$, the HMM form interface state with silver at wavelength 558 nm, so we can observe reflection deep in the KR configuration as shown in Supplementary Fig. S**6** (b) but no reflection deep in the Otto configuration as shown in Supplementary Fig. S**6** (a). The series of reflection deeps < 480 nm and $> 600nm$ result from the excitation of bulk states.

6 Transverse Optical Spin angular Momentum

We follow the description in the main text to calculate the transverse optical spin angular momentum (SAM) density of surface plasmon on the interface between dielectric and metal as shown in Supplementary Fig. S**7**. As shown in Supplementary Fig. S**7**(a) and S**7**(b), the surface plasmons propagate to the x direction and the corresponding transverse SAM is locked to $-y$ and $+y$ direction, respectively. If we compare the SAM of the interface of 1DHMM with that of surface plasmon, we can observe that the tranverse SAM in these cases share similar feature: for the case of $\omega < \omega^{XC}$ (Fig.5(c)), the transverse SAM of the interface state is locked to the $+y$ direction, as same as the case shown in Supplementary Fig. S**7**(d); for the case of $\omega > \omega^{XC}$ (Fig.5(b)), the transverse SAM of the interface state is locked to the $-y$ direction, as same as the case shown in Supplementary Fig. S**7**(c). The SAM flipping during the phase transition verifies that the HMM change from metallic-like to dielectric-like.

References

[1] Atala, M. *et al.* Direct measurement of the zak phase in topological bloch bands. *Nat Phys* **9**, 795–800 (2013).

[2] Archambault, A., Marquier, F. m. c., Greffet, J.-J. & Arnold, C. Quantum theory of spontaneous and stimulated emission of surface plasmons. *Phys. Rev. B* **82**, 035411 (2010).

[3] Landau, L. *et al. Electrodynamics of Continuous Media* (Butterworth-Heinemann, 1984).

[4] Raman, A. & Fan, S. Photonic band structure of dispersive metamaterials formulated as a hermitian eigenvalue problem. *Phys. Rev. Lett.* **104**, 087401 (2010).
